# The Role of Ceramide Metabolism and Signaling in the Regulation of Mitophagy and Cancer Therapy

**DOI:** 10.3390/cancers13102475

**Published:** 2021-05-19

**Authors:** Megan Sheridan, Besim Ogretmen

**Affiliations:** Hollings Cancer Center, Department of Biochemistry and Molecular Biology, Medical University of South Carolina, Charleston, SC 29425, USA; sheridam@musc.edu

**Keywords:** cancer, ceramide, sphingolipids, mitophagy, apoptosis

## Abstract

**Simple Summary:**

Sphingolipids are membrane-associated lipids that are involved in signal transduction pathways regulating cell death, growth, and migration. In cancer cells, sphingolipids regulate pathways relevant to cancer therapy, such as invasion, metastasis, apoptosis, and lethal mitophagy. Notable sphingolipids include ceramide, a sphingolipid that induces death and lethal mitophagy, and sphingosine-1 phosphate, a sphingolipid that induces survival and chemotherapeutic resistance. These sphingolipids participate in regulating the process of mitophagy, where cells encapsulate damaged mitochondria in double-membrane vesicles (called autophagosomes) for degradation. Lethal mitophagy is an anti-tumorigenic mechanism mediated by ceramide, where cells degrade many mitochondria until the cancer cell dies in an apoptosis-independent manner.

**Abstract:**

Sphingolipids are bioactive lipids responsible for regulating diverse cellular functions such as proliferation, migration, senescence, and death. These lipids are characterized by a long-chain sphingosine backbone amide-linked to a fatty acyl chain with variable length. The length of the fatty acyl chain is determined by specific ceramide synthases, and this fatty acyl length also determines the sphingolipid’s specialized functions within the cell. One function in particular, the regulation of the selective autophagy of mitochondria, or mitophagy, is closely regulated by ceramide, a key regulatory sphingolipid. Mitophagy alterations have important implications for cancer cell proliferation, response to chemotherapeutics, and mitophagy-mediated cell death. This review will focus on the alterations of ceramide synthases in cancer and sphingolipid regulation of lethal mitophagy, concerning cancer therapy.

## 1. Introduction

Advancements in cancer therapies and early detection have allowed mortality rates for many cancers to drop or remain consistent over the past 30 years. The most common cancer type to afflict men is prostate cancer and women is breast cancer, with colorectal and lung cancer following next in incidence for both demographics [1]. While improvements have been made on multiple fronts, cancer incidence is still increasing for kidney, pancreas, liver, melanoma, and head and neck cancer [2,3]. Survival rates also remain low for pancreas, liver, lung, and esophageal cancers [1]. To further facilitate innovations to treat rising cancer incidence and reduce mortality, understanding the underlying mechanisms involved in tumor generation, metastasis, and drug resistance is needed. Recent studies have demonstrated the involvement of sphingolipids in tumorigenesis and metastasis. Sphingolipids are membrane lipids that play an important role in disease pathogenesis and the signal transduction of multiple cellular pathways. Notably, the dichotomy between sphingosine-1-phosphate and ceramide has become a point of interest concerning the roles of sphingolipids in cancer. Sphingosine-1-phosphate is generally increased in cancer cells and aids in cell survival and chemotherapeutic resistance. Endogenous ceramide is commonly decreased in cancer cells and acts as an inducer for apoptosis, mitophagy, and necroptosis [4,5].

Mitophagy is a form of selective macro-autophagy that targets depolarized mitochondria for degradation by engulfment in double-membraned autophagosomes and fusion with the lysosome. Recently, it has become apparent that lethal mitophagy plays a role in suppressing tumorigenesis and could provide an avenue for the treatment of various cancers. This process is mediated by ceramide, which accumulates on depolarized mitochondria and can directly bind autophagy marker light chain 3 (LC3II), a marker for autophagosomes [6] (p. 18). Ceramide and sphingosine analogue drugs, such as pyridinium-ceramide and Fingolimod/Gilenya (FTY720), have demonstrated the antiproliferative effects of targeting the sphingolipid pathway in cancer. This review will focus on the mechanism of action of ceramide signaling in the regulation of mitophagy and tumor suppression with regards to cancer therapy.

## 2. Sphingolipids in Cancer

Cellular stress induces the generation of sphingolipids that mediate complex processes such as apoptosis, necroptosis, lethal mitophagy, and senescence which combat transformation. Cancer cells can undermine these protective processes by dysregulating the enzymes involved in sphingolipid metabolism. Cancer cells can further support their proliferation, metastasis, and resistance to chemotherapeutics by upregulating the production of pro-survival sphingolipids, such as sphingosine-1-phosphate and downregulate pro-cell death sphingolipids like ceramide.

### 2.1. Sphingolipid Structure and Metabolism

**Figure 1:**
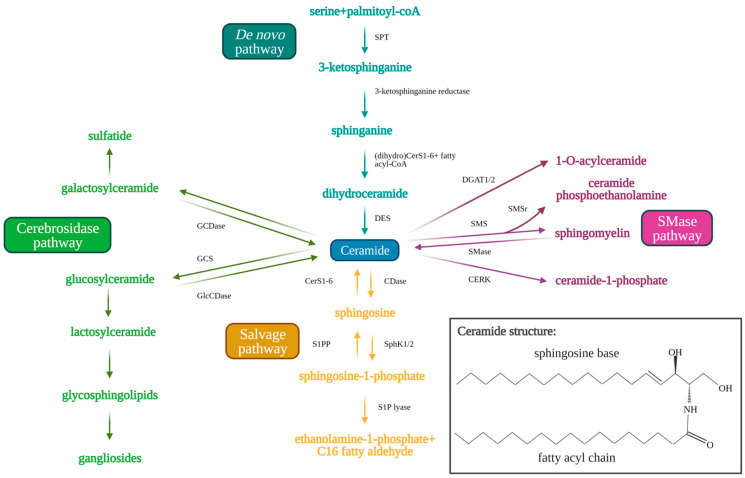
Sphingolipid metabolism pathway. Ceramide acts as the central molecule of the sphingolipid metabolism pathway. Ceramide may be synthesized through either the de novo (blue text and arrows) or the salvage pathway (orange text and arrows). Synthesis of ceramide through the de novo pathway occurs through serine palmitoyltransferase (SPT), 3-ketosphinganine reductase, (dihydro)CerS1-6 (which determine fatty acyl chain length), and finally dihydroceramide desaturase (DES). Ceramide synthesis through the salvage pathway occurs through conversion of sphingosine-1-phosphate by S1P-phosphatase (S1PP) and (dihydro)CerS1-6. Ceramide may be metabolized to produce sphingosine 1-phosphate by ceramidases (CDases) and sphingosine kinase 1/2 (SphK1/2). Sphingosine-1-phosphate can be hydrolyzed by sphingosine 1-phosphate lyase (S1P lyase) to ethanolamine 1-phosphate and C_16_ fatty aldehyde. Ceramide may also be used as a substrate for the generation of complex sphingolipids via conversion to glucosylceramide (GlcCer) by glucosylceramide synthase (GCS) (green arrows and text). Glucosylceramidase (GlcCDase) and galactosylceramidase (GCDase) (cerebrosides) catalyze conversion of complex sphingolipids back to ceramide. Ceramide may also be used as a substrate for sphingomyelin through sphingomyelin synthase (SMS) or ceramide 1-phosphate through ceramide kinase (CERK). Sphingomyelin can similarly be broken down by sphingomyelinase (SMase) to produce ceramide once again. Created with BioRender.com (accessed on 11 May 2021).

Sphingolipids are bioactive cell membrane molecules that play important roles in signal transduction pathways such as controlling cell death/survival, proliferation, migration, and senescence. Treatment of cancer cells via chemotherapy, radiotherapy, or with anticancer drugs (such as cisplatin) that produce oxidative stress, generates ceramide and sphingosine. Ceramide is a pro-apoptotic sphingolipid that consists of a long-chain sphingosine base and an amide-linked fatty acyl chain that varies from 14 to 26 carbons in length [7,8,9,10]. Endogenous ceramides are produced by the de novo synthesis pathway (Figure 1) in the endoplasmic reticulum, which first consists of serine and palmitoyl CoA condensation to 3-keto-sphinganine by serine palmitoyltransferase (SPT) [11,12,13,14]. SPT is a multi-subunit enzyme that may be negatively regulated by orosomucoid (ORM)-like protein 1-3 (ORMLD1-3) (particularly ORMLD3) through stable interaction with small activating SPT subunits to inhibit downstream ceramide synthesis [15,16,17]. The 3-ketosphinganine is then reduced by 3-ketosphinganine reductase to produce sphinganine, which is used by (dihydro)ceramide synthases (CerS1-6) in a rate-limiting reaction to yield dihydroceramide via the addition of the variable fatty acyl chain [18,19]. Dihydroceramide desaturase (DES) then inserts a trans-double bond in dihydroceramide between carbons 4 and 5 of the long-chain sphingosine base to produce endogenous ceramide [7,19]. Ceramides generated by CerS1–6 have different fatty acyl chain lengths, which affects their cellular functions. CerS1 and CerS4 generate C18–C20 ceramides, CerS5 and CerS6 generate C14–C16 ceramides, CerS2 generates longer C22–C24 ceramides, and CerS3 generates ultra-long chain C28–C32 ceramides specific to the skin and testes [19,20,21,22]. Ceramide may be alternatively produced by the salvage pathway, which recycles sphingosine and exogenous short chain ceramides (C2–C6) to generate endogenous long chain ceramides (C14–C26) using ceramide synthases (CerS1–6) essential to de novo synthesis [21]. Ceramide is also produced from sphingomyelin hydrolysis by sphingomyelinases (SMases) or from glucosylceramide and/or galactosylceramide breakdown by glucosylceramidase (GlcCDase) and galactosylceramidase (GCDase), respectively [7,9,10,22].

Ceramide may also be used as a substrate to produce other sphingolipids. Ceramide can be hydrolyzed by ceramidases (CDases), such as acid ceramidase to produce sphingosine [10,23]. Sphingosine kinase 1 (SphK1) or 2 (SphK2) phosphorylates sphingosine to yield sphingosine-1-phosphate (S1P). S1P has been shown to induce opposing pro-survival effects compared to ceramide, promoting cell proliferation, migration, and growth. S1P induces these effects by interacting with transmembrane G-protein coupled receptors, S1P receptors 1-5 (S1PR1-5), in an autocrine or paracrine manner [10,23]. S1P is then rapidly metabolized in 10–20 min by S1P phosphatase or S1P lyase to produce ethanolamine 1-phosphate and C16-fatty aldehyde [24,25]. Alternatively, ceramide may be used as a substrate by glucosylceramide synthase (GCS) to produce glucosylceramide (GlcCer), which is moved from the early to distal Golgi compartment by phosphatidylinositol-4-phosphate adaptor protein 2 (FAPP2) [14,26]. GlcCer then assists in the synthesis of lactosylceramide and finally complex glycosphingolipids which contributes to the lipid composition of the plasma membrane [26]. Ceramide may also be used by sphingomyelin synthase (SMS) to produce sphingomyelin via the insertion of phosphorylcholine from phosphatidylcholine. SMS-related protein (SMSr) has similarly been shown to catalyze the conversion of ceramide to trace ceramide phosphoethanolamine. Knockdown of SMSr dramatically increased ER-associated ceramide levels, which resulted in fragmentation and Golgi collapse [27]. Additionally, ceramide kinase (CERK) converts ceramide to ceramide-1-phosphate (C1P), and diacylglycerol acyltransferase (DGAT1/2) converts ceramide to 1-O-acylceramide [28,29,30]. The sphingolipid metabolic pathway is shown in Figure 1 below.

### 2.2. Sphingosine Kinase 1 and 2

S1P has a pro-survival effect on cancer cells, increasing cellular fitness, invasion, and metastasis. In many cancers, S1P production and/or secretion is increased, indicating that antagonism of S1P may provide a potential treatment option for patients. While both SphK1 and SphK2 produce S1P, these kinases have different downstream signaling targets that are affected by SphK1′s localization in the cytosol and SphK2′s localization in the nuclear membrane and cytoplasm [10]. Patients afflicted with cancers that had increased SphK1 mRNA expression were found to have worse prognosis and survival outlooks [31]. Notably, SphK1 was found upregulated in many different cancers, such as kidney cancer, prostate cancer, liver cancer, colorectal cancer, gastric cancer, uterine cancer, ovarian cancer, lung cancer, breast cancer, lymphoma, glioblastoma, small bowel cancer, and myeloid leukemia [32,33,34,35]. For example, many colon cancers exhibit overexpression of SphK1, and a SphK1 knockout mouse model that was exposed to azoxymethane developed less colon tumors than wild-type mice [36]. A similar effect was found in triple-negative breast cancer (TNBC), which has overexpressed S1P [37,38,39]. Exogenous SphK1 and SphK1 overexpression was also found to induce migration, proliferation, and invasion of ovarian cancer cells in vitro. SphK1 overexpression increased tumor burden in a mouse xenograft model [40]. As solid tumors develop, vascularization is often induced to provide nutrients to internal cancer cells. Inhibition of SphK1 or S1PR1/3 reduced angiogenic factor secretion in epithelial ovarian cancer and clear cell renal cell carcinoma [41,42,43]. Inhibition of SphK1 using FTY720 or SKI-II in cervical cancer lines reduced the expression of matrix metalloproteinase-2 and vascular endothelial growth factor-A, attenuating invasion and angiogenesis [44].

FTY720 (Fingolimod/Gilenya, Novartis, Basel, Switzerland) is a sphingosine analogue drug that is phosphorylated by SphK2 to produce a structural analogue of S1P and functional antagonist for S1PR1 that acts a tumor suppressor in colon and lung cancer cell lines and mouse models [45,46,47,48]. FTY720 has been shown to inhibit chronic myeloid leukemia stem cell proliferation, and successfully suppressed human lung cancer in a xenograft mouse model at physiologically relevant concentrations [47,49]. Similarly, SK1-I is a sphingosine analogue and competitive inhibitor of SphK1, while SK1-II is a competitive inhibitor of both SphK1 and SphK2. SK1-I has been shown to inhibit glioblastoma proliferation in cell lines and xenograft mouse models [35,50]. Similarly, PF-543 is a potent selective SphK1 inhibitor which induces the proteasomal degradation of SphK1. It was found that PF-543 induces colorectal cancer cell death both in vitro and in vivo [51]. ABC294640 (Opaganib, Red Hill, Tel-Aviv, Israel) is a SphK2 inhibitor that has been shown to suppress lung and pancreatic cancer cell growth, along with attenuation of cell senescence via inhibition of telomerase stability through telomere damage response [52,53]. ABC294640 has been utilized in a number of clinical trials for the treatment of various cancers such as sorafenib resistant hepatocellular carcinoma, refractory multiple myeloma, diffuse large B-cell lymphoma, Kaposi sarcoma [54,55,56], and prostate cancer.

Like SphK1, SphK2 has been found to be overexpressed in various cancer types such as non-small-cell lung cancer and colorectal cancer [57,58]. SphK2 overexpression was associated with lower patient survival and gefitinib resistance [57]. It was reported that the ERK1 phosphorylation of SphK2 in an epidermal growth factor-mediated manner stimulated breast cancer cells to migrate in vitro [59]. Cancers also commonly upregulate human telomerase reverse transcriptase (hTERT) to prevent cell senescence induced by their high proliferation rate and degradation of chromosomal telomeres. SphK2 generated S1P has been found to stabilize hTERT by mimicking phosphorylation via binding hTERT involving its Asp648. Knocking down or inhibiting SphK2 inhibited growth of lung cancer cells in vitro and in mouse xenograft models [52]. These studies have further shown that antagonizing S1P signaling could provide therapeutic benefit to patients suffering from many different cancer types.

### 2.3. Ceramide Synthases 1–6

CerS1-6 function in the production of variable length ceramides that have distinct effects on cancer cell growth and survival. CerS1 is notable for its synthesis of C18-ceramide, a pro-apoptotic and anti-tumorigenic ceramide that has been shown to be downregulated in head and neck squamous cell carcinoma (HNSCC) compared to healthy control tissue [60,61,62,63,64]. C18-ceramide production by CerS1 is inhibited in HNSCC through transcriptional repression via histone deacetylase 1 (HDAC1)-dependent inhibition of Sp1 at the promoter, and post-transcriptionally by miR574-5p that induces translation of a CerS1 isoform 2 splice variant common in HNSCC tumor tissues [65]. Reduced C18-ceramide levels in HNSCC has been associated with tumor metastasis, advanced stage cancer, and increased lymphovascular invasion [61,62,66]. C18-ceramide has also been shown to interact with inhibitor 2 of protein phosphatase 2A (I2PP2A), which indirectly activates PP2A and induces degradation of c-Myc in lung adenocarcinoma cell lines [47,67,68,69,70,71]. CerS1 depletion has been associated with poorer prognosis and patient outcomes in breast cancer and neuroblastoma [64,72]. Reduced levels of C18-ceramide were also found in colorectal cancer and glioma tissue, where exogenous C18-ceramide was found to induce ER-stress and lethal autophagy in the latter [73,74]. Like CerS1, CerS4 also contributes to C18-C20 ceramide synthesis. CerS4 mRNA was found to be higher in early stage, non-metastatic HNSCC, melanoma, and renal cell carcinoma tumors compared to later stage, aggressive, metastatic tumors. Knocking down CerS4 in lung adenocarcinoma and HNSCC cell lines reduced cell migration in vitro and liver metastasis from murine mammary cancer cells in vivo [75].

CerS2 generates longer C22–C24 ceramides and was similarly found to be downregulated in many cancers. Mice that expressed a catalytically inactive mutant CerS2 developed hepatocellular carcinoma (HCC) at a young age (8 weeks), while mice that were deficient for CerS2 developed liver adenoma and HCC later in adulthood (7–10 months) [76,77,78]. Similarly, CerS2 knockout mice were susceptible to azoxymethane induction of colon carcinoma and dextran sodium sulfate induction of colitis [36]. CerS2 overexpression in MDA-MB-231 breast cancer cells was shown to decrease cell migration and invasion, and CerS2 expression was associated with improved patient survival in breast, ovarian, lung, and liver cancer [65,79]. CerS5 has been noted to be upregulated in colorectal cancer and was associated with poor patient survival and 5-year cancer recurrence [73,80,81]. CerS5 has also been noted to be a biomarker for colorectal cancer [81]. Interestingly, CerS5 and CerS6 knockdown was shown to sensitize mice to azoxymethane/dextran sodium sulfate-induced colitis and increased cases of colitis-associated colon cancer [82,83]. CerS6 is notable for its generation of C16-ceramide, which is involved in apoptosis induction and the protection of ER and Golgi membrane integrity in cancer. CerS6 derived C16-ceramide was found to induce apoptosis in lung adenocarcinoma cells following non-genotoxic folate stress through p53 transcriptional targeting [84]. C16-ceramide induced apoptosis through BAX in HeLa cells following irradiation, and C16-ceramide also activated caspase 3 translocation to the nucleus resulting in increased tumor necrosis factor-related apoptosis-inducing ligand (TRAIL) sensitivity in colon cancer cells [85,86]. Paradoxically, C16-ceramide was found elevated in oral and gastric cancer [64,87,88]. CerS6 overexpression has been described as a biomarker in gastric cancer, and CerS6 overexpression was associated with poor patient survival, invasion, and metastasis in gastric cancer [88]. It was found that HNSCC exhibited upregulated CerS6 derived C16-ceramide, which resulted in a protective effect seen towards the ER and Golgi membrane integrity. When CerS6 was inhibited, activating transcription factor 6 (ATF6) induced ER-stress resulting in cell death [10,87,89,90]. Overall, these studies suggest that distinct/paradoxic biological roles of ceramides in cancer are context dependent regulated by their subcellular localization and down-stream targets.

## 3. Autophagy and Mitophagy in Cancer

One of the downstream biological responses that ceramide signaling exerts is induction of autophagy and mitophagy. These processes are essential cellular processes to maintain tissue and cellular homeostasis by preventing the accumulation of damaged organelles and reactive oxygen species that can cause systemic inflammation and create a pro-tumorigenic environment. While autophagy and mitophagy may have tumor suppressing effects, cancers are able to modulate the activity of these pathways to suit tumor growth and metastasis.

### 3.1. Mechanism of Autophagy and Mitophagy

**Figure 2:**
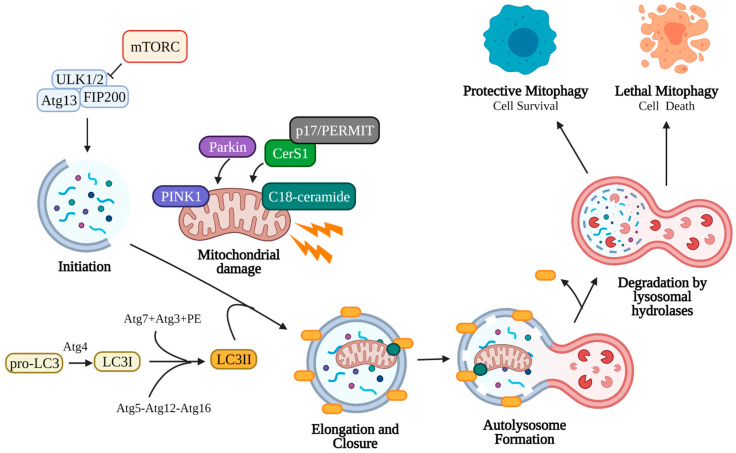
Canonical mitophagy pathway. Damaged mitochondria signal the need for removal by retaining PINK1 on the outer mitochondrial membrane, which recruits cytosolic Parkin and p62. Parkin ubiquitinates outer mitochondrial membrane proteins, allowing p62 recruitment and direct binding to LC3II. LC3II is an essential component of the autophagosome double-membrane and regulates its elongation and curvature. LC3II is formed following cleavage of the C-terminal by Atg4, and LC3I is subsequently conjugated to phosphatidylethanolamine (PE) by Atg7, Atg3, and the Atg5-Atg12-Atg16 complex. Elongation of the autophagosomal membrane occurs around the mitochondria until it is fully engulfed. The autophagosome is then trafficked to the lysosome, where the outer autophagosomal membrane fuses with the lysosome. Internal components, including the mitochondria, are degraded and recycled by lysosomal hydrolases (cathepsin B, D, L). Mitophagy may assist the cell in reducing stress (protective mitophagy resulting in cell survival) or in inducing cell death (lethal mitophagy). Created with BioRender.com (accessed on 11 May 2021).

Autophagy is the process by which organelles and cellular components are degraded by the lysosome and macromolecules and nutrients are recycled by the cell (Figure 2). This process may occur through micro-autophagy, chaperone-mediated autophagy, or macro-autophagy. Micro-autophagy involves the degradation of bulk cytosolic contents by the lysosome [91,92]. Chaperone-mediated autophagy proceeds via binding of Hsc70 chaperone to substrates containing a KFERQ motif, which then binds LAMP2A on lysosomes for internalization and degradation [93]. Macro-autophagy (referred to here as autophagy) involves the degradation of cellular components through autophagosome engulfment and fusion with the lysosome. This process may be non-selective (bulk degradation induced by starvation) or selective (specific organelles) [91].

Autophagy begins with the initiation phase, where a cup-shaped double-membrane called a phagophore (also called an isolation membrane) begins to form. The phagophore is induced by the Unc-51-like kinase 1/2 ULK1/2 complex, which activates PI3K complex 1 to mediate the nucleation of the phagophore membrane [91,94]. Classic induction of autophagy entails starvation or rapamycin treatment to inhibit target of rapamycin complex 1 (mTORC1) kinase, which allows ULK1/2 to phosphorylate autophagy related protein 13 (Atg13) and family interacting protein of 200 kD (FIP200) to form the initiation complex and begin autophagic process [95,96,97,98,99]. Next, the elongation phase begins to extend the phagophore membrane around the target cell component. This elongation occurs when microtubule-associated protein 1A/1B-light chain 3 (LC3, the mammalian homolog of yeast Atg8) and Atg5-Atg12-Atg16 (Atg16 complex) are recruited to the phagophore [91,94,100]. The C-terminal of cytosolic pro-LC3 is cleaved by Atg4 cysteine protease, yielding LC3I. LC3I is then conjugated to phosphatidylethanolamine (PE) in a ubiquitin-like reaction by Atg7, Atg3, and finally Atg12-Atg5, forming LC3II [96,101,102]. Elongation continues until the target organelle has been completely enclosed by the double membrane (closure), forming a mature autophagosome [103,104,105,106]. LC3II is an essential component of the autophagosome membrane, controlling membrane length and curvature, and acts a marker for autophagosomes. The autophagosome containing the cell material is trafficked to the lysosome, where the outer membrane fuses with the lysosome forming an autolysosome. To conserve LC3, Atg4 cleaves PE from LC3II on the outer autophagosomal membrane and releases it back into the cytoplasm [96]. The inner autophagosomal membrane and its contents are degraded by lysosomal hydrolases (cathepsin B, D, L), and the macromolecular precursors (amino acids, lipids, etc.) are released back into the cytoplasm for use in other cellular processes [107,108]. The rate at which autophagosomes are formed and degraded in this manner is called autophagic flux.

The selective macro-autophagy of mitochondria is called mitophagy, which is an essential cellular process that allows recycling of damaged mitochondria. Damaged mitochondria that are not removed by mitophagy risk the leakage of reactive oxygen species, Ca^2+^, and cytochrome c into the cytosol, inducing apoptosis [109,110,111,112]. Since mitochondria exist in an interconnected network, damaged mitochondria must first be sequestered for engulfment by autophagosomes [113,114]. This can occur via fission through the action of dynamin-related protein 1 (Drp1), which forms a multimeric complex around the mitochondrion and exerts mechanical force to separate it from the mitochondrial network [115,116,117]. Depolarization of the mitochondrial membrane from damage also induces fragmentation of the mitochondria due to the loss of surface fusion proteins, such as optic atrophy 1 (Opa1) and mitofusin 1 and 2 (Mtf1, Mtf2) [112,118,119,120,121]. Depolarized mitochondria signal the need for mitophagy by preventing the import of PTEN-induced kinase 1 (PINK1), which accumulates on the outer mitochondrial membrane [122,123,124,125,126]. PINK1 accumulation induces translocation of cytosolic Parkin (E3-ubiquitin ligase) to the mitochondria, where Parkin deposits lysine 48 (K48) and K63 ubiquitin chains to initiate proteasomal degradation of outer membrane proteins such as mitofusin, preventing fusion [121,126,127]. P62, a ubiquitin binding protein, translocates and accumulates on depolarized mitochondria, allowing direct binding to autophagosomal LC3II and engulfment in autophagosomes [128,129,130] (Figure 2).

### 3.2. Autophagy Paradox and Cancer

Autophagy and mitophagy play an important role in promoting cancer cell survival or mediating cancer cell death, which are context/cell type dependent. Interestingly, autophagy repression or activation can have pro- or anti-survival effects at different stages of cancer development, referred to as the autophagy paradox. At the onset of carcinogenesis, autophagy may be activated in a tumor-suppressive manner to combat viral oncogenes, nutrient deprivation, increased oxidative stress, and DNA damage stemming from the higher proliferation rate in cancer cells [131,132]. As a result, some cancers downregulate autophagy in early stages [133]. However, once a tumor has become established, the faster rate of proliferation and subsequent nutrient deprivation may motivate the cancer to activate autophagy and increase its autophagic flux [128]. As the interior of the tumor environment becomes more hypoxic, autophagy may also assist cancer cells in resisting environmental stress [115]. Many aggressive cancers have been documented to have elevated autophagic flux, such as pancreatic cancer, head and neck squamous cell carcinoma, non-small cell lung cancer, and colorectal cancer [134,135,136]. As a result, treating these more advanced stage cancers with autophagy inhibitors has proven beneficial in inducing apoptosis and senescence in vitro and in vivo.

Mitophagy regulation is important in cancers as a failure to remove depolarized mitochondria results in the release of reactive oxygen species and cytochrome c to induce apoptosis. Similar to general autophagy, mitophagy may be modulated to assist in cancer cell survival. It is notable that many cancer cells alter their generation of adenosine 5′-triphosphate (ATP) from mitochondrial oxidative phosphorylation to aerobic glycolysis to produce lactate and nutrients needed for rapid proliferation, a phenomenon called the Warburg effect [109,137,138,139,140]. This effect is supplemented by the prevalence of KRAS-proto-oncogene, GTPase (KRAS) mutations in cancers, which upregulates glycolysis but strains mitochondria [108,141,142]. As a result, rapid mitochondrial turnover (mitophagy) is needed to accommodate growth and glycolysis. This need for rapid mitophagy is demonstrated in adenomas and oncocytomas, where more aggressive adenomas that become defective in autophagy may form less aggressive oncocytomas, which are abundant in damaged mitochondria [143]. Mitophagy upregulation at the onset of carcinogenesis can be anti-tumorigenic. Dysfunctional mitochondria and ablated mitophagy puts cells at risk of transformation. PINK1 and Parkin deletion in mice led to the development of spontaneous hepatocellular carcinoma and KRAS-induced pancreatic cancer development, while a mouse model of breast cancer that was knocked down for BCL2/adenovirus E1B 19 kDa protein-interacting protein 3 (BNIP3) to reduce mitophagy supported breast tumor progression [144,145,146].

Nevertheless, reactive oxygen species (ROS) production due to a hypoxic tumor microenvironment, high proliferation rate, dysfunctional mitochondria accumulation, and downregulation of pro-apoptotic factors may result in elevated levels of ROS in cancer cells. To combat increased ROS levels, cancer cells upregulate antioxidants such as manganese-superoxide dismutase. Autophagy, specifically mitophagy, is also increased to prevent leakage of superoxide from the electron transport chain, which produces hydrogen peroxide. This results in an increased sensitivity to exogenously induced ROS in many cancer cells [83,147]. ROS production was found to induce autophagy in human glioma cells treated with electron transport chain inhibitors rotenone and thenoyltrifluoroacetone (TTFA). Knockdown of superoxide dismutase 2 in glioma cells increased autophagy in rotenone and TTFA treated cells [148]. Colorectal cancer cells treated with ciclopirox olamine (CPX) was similarly found to downregulate Parkinsonism associated deglycase (PARK7, DJ-1), which increased ROS production and mitochondrial dysfunction to induce mitophagy in a pro-survival manner [149]. SMAD4 knockdown desensitized pancreatic cancer cells to radiotherapy through induction of ROS and protective autophagy [150].

### 3.3. Ceramide Mediated Mitophagy

**Figure 3:**
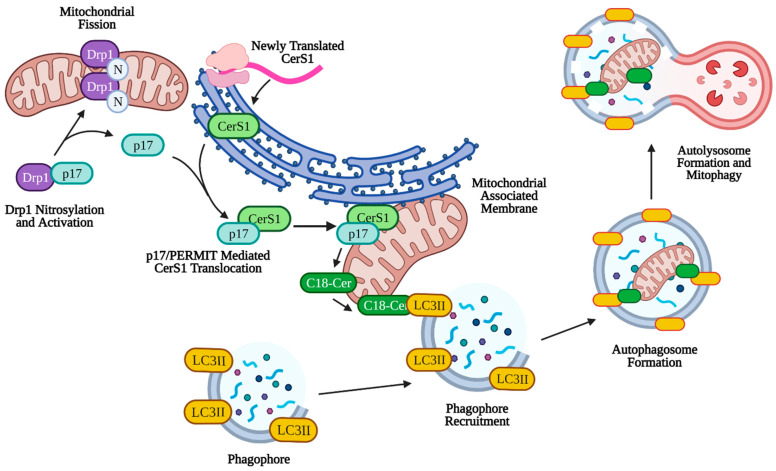
p17/PERMIT-mediated mitophagy. C18-ceramide induced mitophagy is facilitated by p17/PERMIT translocation of CerS1 to the mitochondria. Drp1 is activated by S-nitrosylation at C644, which releases p17/PERMIT and initiates mitochondrial fission. P17/PERMIT associates with newly translated CerS1 at the mitochondrial-associated membrane and is translocated via a mitochondrial signaling sequence (amino acids L21–R25). CerS1 is then able to produce C18-ceramide, which coats the outer mitochondrial membrane and directly binds autophagosomal LC3II, facilitating mitophagy. Created with BioRender.com (accessed on 11 May 2021).

Mitophagy can play a larger role in tumor suppression through lethal mitophagy, the process by which mitochondria are degraded via mitophagy to the extent that the cell dies in an apoptosis-independent manner. Simple upregulation of mitophagy or sustained mitophagy over long periods of time leads to caspase-dependent apoptosis through leakage of cathepsin proteases from the lysosome [151,152,153]. Lethal mitophagy is mediated, in part, by CerS1 and its product, C18-ceramide and is independent of caspase 3, Bax, and Bak. It was shown that CerS1-generated C18-ceramide and an exogenous C18-pyridinium-ceramide analogue accumulated on the outer mitochondrial membrane due to the positive charge associated with the pyridinium head group. This accumulation of ceramide on mitochondria allowed C18-ceramide to directly bind the hydrophobic domain (specifically Ile35 and Phe52) of LC3II on the phagophore to facilitate enclosure of the mitochondria by the autophagosome [6]. Lethal mitophagy was shown to inhibit HNSCC and acute myeloid leukemia both in vitro and in vivo [154]. It was found that C18-ceramide mediated lethal mitophagy was distinct from survival autophagy, induced by cell starvation, in HNSCC. Lethal mitophagy progressed following dynamin-related protein 1 (DRP1) fission of mitochondria involving protein kinase A (PKA) inhibition and reduced phosphorylation of Fms-like tyrosine kinase 3 (FLT3) in acute myeloid leukemia cells from patients and immunocompromised mice [154] (p. 3). Interestingly, CerS6 derived C16-ceramide was not effective in inducing lethal mitophagy or binding LC3II, but artificial accumulation on the mitochondria using a C16-ceramide analog with a positively charged head group was effective in inducing lethal mitophagy, indicating that the length of the fatty acyl chain may not have as much of an effect as subcellular location of ceramide on the outer mitochondrial membrane [6,155]. More specifically, it was discovered that endogenous C18-ceramide accumulation on the outer mitochondrial membrane was mediated by the translocation of newly translated CerS1 from the ER by protein that mediates ER-mitochondria trafficking (p17/PERMIT) (Figure 3). Under conditions of cell stress, p17/PERMIT dissociates from Drp1 following Drp1 S-nitrosylation and activation in the cytoplasm, which induces mitochondrial fission (see above). P17/PERMIT is then able to retrieve newly translated CerS1 from ER-mitochondrial contact sites called mitochondrial-associated membranes (MAMs), allowing the generation of C18-ceramide on the outer mitochondrial membrane which induces mitophagy [156]. Therefore, C18-ceramide mediated lethal mitophagy may be considered a potent anti-tumorigenic mechanism (Figure 3).

Mitophagy and autophagy may be activated and regulated through alternative pathways in cancer as well. Inhibition of ceramide transfer protein (CERT), which transports ceramide from the endoplasmic reticulum to the Golgi, was found to cause accumulation of hexosylceramide on mitochondria, increased reactive oxygen species, and increased mitophagy, resulting in premature cell senescence [157]. Isc1p, a yeast ortholog of mammalian neutral sphingomyelinase-2 (N-SMase2), is transported from the endoplasmic reticulum to the mitochondria during respiratory metabolism. Knockdown of Isc1p upregulated Dnm1p-mediated mitochondrial fission and subsequent mitophagy while ceramide signaling through Sit4p and Hog1p kinase was activated (supporting mitophagy) [158]. Mitophagy not only has an impact on cancer cell survival and invasion, but also on the immune cells necessary for tumor clearance. Suppression of C3aR/C5aR activation induced lethal mitophagy in dendritic cells and suppressed graft-versus-host disease following hematopoietic cell transplantation [159].

Similar to p17/PERMIT regulation of mitophagy through the transport of CerS1, general autophagy was found to be modulated via translocation of ceramide-1-phosphate from the trans-Golgi network by ceramide-1-phosphate transfer protein (CPTP). Downregulation or mutational inactivation of CPTP induced autophagy, along with trans-Golgi network fragmentation and inflammasome activation in acute monocytic leukemia cells [160]. Caveolin-1, which is upregulated in prostate cancer and acts as a lipid chaperone in caveolae, is associated with a more aggressive cancer phenotype, tumor metastasis, and chemotherapeutic resistance. Caveolin-1 was reported to alter ceramide metabolism, scavenging extracellular sphingomyelin to skew production towards glycosphingolipids and inhibition of mitophagy [161]. Studies examining the role of autophagy and mitophagy in cancer treatment have led to the development of drugs targeting this pathway for tumor clearance as well. ABTL0812, a drug undergoing analysis in phase 2 clinical trials for advanced endometrial cancer and squamous non-small cell lung cancer, induced ER stress and autophagy through upregulation of long-chain dihydroceramides in many cancer cell types [162]. The addition of an oxazoline ring to doxorubicin and daunorubicin overcame drug resistance in ovarian and hepatocellular cancer cell lines through increased neutral sphingomyelinase and autophagy induction [163]. Ceramides may even be incorporated into treatments, as with C6-ceramide-tamoxifen treatment of acute myeloid leukemia, which promotes cancer cell death through lethal mitophagy [164].

In contrast to ceramide-mediated autophagy/mitophagy and cancer cell death, S1P metabolism and signaling appear to prevent autophagy-associated cell death in various cancer types. For example, it has been shown recently that inhibition of SphK1 by SK1-I results in autophagy-mediated cell death via ATG5 and BECN-1, which is dependent on p53 in HCT116 human colon cancer cells [165]. Moreover, inhibition of SphK2 using ABC294640 was reported to induce autophagy-mediated cell death and tumor suppression in Kaposi sarcoma-associated herpes virus-related tumors [166].

Overall, these studies support a role for ceramide/S1P metabolism and signaling in the regulation of autophagy and/or mitophagy for controlling cancer growth and therapy.

## 4. Sphingolipids and Cancer Therapy

### 4.1. Cancer Therapy and Drug Resistance

As mentioned previously, many cancers have reduced levels of pro-apoptotic C18-ceramide generated by CerS1 while pro-survival S1P production is upregulated. Cellular levels of C18-ceramide may be reduced through the inhibition of CerS1/4 or by an increase in the level of ceramide metabolizing enzymes (see above). As a result, chemotherapeutic drugs and radiotherapy serve to sensitize cancer cells and induce apoptosis by restoring ceramide levels. For example, in a phase II clinical trial, some patients receiving gemcitabine and doxorubicin for the treatment of head and neck cancers had elevated serum C18-ceramide [10,63]. These patients were reported to have better clinical outcomes (partial response, complete response, or stable disease state) than those with normal serum C18-ceramide (progressing disease state) [63]. These results were confirmed in HNSCC cell lines and mouse xenograft tumors that showed a CerS1-dependent increase in C18-ceramide generation following treatment [60]. Even compounds exhibiting structural similarities to ceramide were noted to exhibit anti-proliferative effects through mitophagy, as was seen with native solenopsin treatment in melanoma cell lines [167]. Similarly, G-protein coupled receptor 1 overexpression induced autophagy and reduced doxorubicin resistance in breast cancer cells through stimulation of the de novo sphingolipid metabolism pathway [168]. Notably, to combat the generation of C18-ceramide by chemotherapeutic drugs, cancer cells may convert ceramide to other sphingolipids in an effort to avoid apoptosis or lethal mitophagy and lessen the potency of the treatment. Ceramide may be hydrolyzed by ceramidases to eventually produce pro-survival S1P or glycosylated by glucosylceramide synthase to produce glucosylceramide, which is associated with chemotherapeutic drug resistance [169,170,171,172,173]. As mentioned in Section 3.2, cancer may upregulate basal mitophagy in a protective manner to reduce reactive oxygen species and restore depolarized mitochondria during chemotherapeutic treatment. Disruption of cancer-induced protective mitophagy through the use of lysosomal inhibitors was found to sensitize drug-resistant HNSCC cells to C6-ceramide nanoliposome delivery of therapeutic ceramide [174]. Conversely, knocking down neutral ceramidase in mouse embryonic fibroblasts stimulated protective autophagy and ceramide generation to protect against drug-induced necroptosis [175].

### 4.2. Immunotherapy and Sphingolipids

Sphingolipids not only exert pro-survival or pro-death effects on cancer cells themselves, but also on the immune cells responsible for mounting cytotoxic responses towards transformed cells. For example, it has been known that S1P/S1PR1 signaling plays essential roles in lymphocyte egress, which is critical for immunity and can be targeted in the treatment of autoimmune disorders [176,177,178,179]. Moreover, sphingolipids have been documented to modulate anti-cancer immunotherapy and immunology in the tumor microenvironment. In melanoma cells, knockdown of SphK1 inhibited the secretion of immunosuppressive cytokines (ex. TGF-β), which sensitized the melanoma cells to anti-PD-1 and anti-CTLA-4 immunotherapies in mice [180,181,182]. S1P secretion from lung cancer cells was also found to impair CD8 positive T cell responses, facilitating metastasis [180]. Patients with bladder cancer were found to have higher levels of S1PR1 and immunosuppressive cytokine secretion (TGF-β, IL-10), along with more circulating and tumor-infiltrating regulatory T cells, which is associated with poor patient outcomes [183]. When S1P secretion was inhibited in mice through knockdown of Spinster Homologue 2 (Spns2), an S1P transporter, pulmonary metastasis of melanoma cells was reduced compared to wild-type mice [184]. Additionally, when SphK1 or S1PR1 was therapeutically targeted or downregulated in mantle cell lymphoma, natural killer cell activation was increased [185]. Notably, ceramide was also found to affect the potency of immunotherapies and T cell responses. C16-ceramide was reported to increase the T cell response to allogeneic hematopoietic stem cell transplantation for the treatment of leukemia in mice. When CerS6 was knocked down in these mice, graft-versus host disease was ablated; an important discovery for patient treatment of leukemia [186]. Recent study demonstrated a role for SphK1/S1P in the attenuation of anti-tumor functions of T cells by inhibiting their bioenergetics. These studies also revealed that targeting SphK1/S1P signaling enhances T cell function against tumors by reprogramming their lipid metabolism, improving immunotherapy for cancer treatment [187,188,189].

## 5. Conclusions and Future Directions

Sphingolipid metabolism, and the generation of S1P and ceramide in particular, have been increasingly investigated concerning their roles in cancer survival, death, and resistance to chemotherapeutics and immunotherapies. Understanding the underlying mechanisms as to how these sphingolipids sensitize or desensitize cancers is essential to the development of new cancer therapies for patients. It has been noted that the subcellular location of ceramide on mitochondria, exogenous or endogenous and regardless of fatty acyl chain length, can induce a potent anticancer effect through the induction of lethal mitophagy [6]. In contrast, localization of S1P to the nuclear membrane, where it can interact with hTERT, increases the survival of aggressive cancers through stabilization of chromosomal telomeres [46]. Targeting these dichotomous sphingolipids with analogue drugs such as FTY720 or pyridinium-C_18_-ceramide has demonstrated that these sphingolipids are important modulators of cancer cell survival pathways.

Additional research is needed to elucidate the complex interactions these sphingolipids have in regulating tumor growth and interaction with the immune environment. Advancements in analytical technology is providing more opportunities to discover these interactions. Without progress in mass spectrometry analysis of sphingolipids, or characterization of the enzymes essential to sphingolipid metabolism, the effect of ceramide and S1P would not have been known. Sphingolipid analog drugs based on these characterizations are providing new therapies for patients, as seen with FTY720, ABC294640, and nanoliposomal ceramides use in clinical trials [42,44,48,49,50,190]. Further research may be made in the regulation of mitophagy through the novel protein p17/PERMIT and the association with mitochondrial and ER-associated membranes [156]. The interactions of secreted and circulating S1P with the tumor microenvironment and immune system could assist in the development of more potent anti-cancer immunotherapies as well. Additionally, interesting research has been published on CerS4 generated C_18_–C_20_-ceramide induction of TGF-β receptor I/II to primary cilia, which increased cancer cell migration and metastasis [74]. Research into these mechanisms and their effects on cancer therapy will provide key insights for the development of patient therapies as analytical and pharmaceutical tools continue to evolve. We believe that targeting sphingolipid metabolism and signaling safely and effectively will also be beneficial for the treatment of other diseases such as neurodegenerative, autoimmune, and aging-associated diseases in the future.
